# Gleanings from Camp and Hospital

**Published:** 1874-06-01

**Authors:** F. K. Bailey

**Affiliations:** Knoxville, Tenn.


### MEDICAL EXAMINER.

A

*Semi-Monthly Journal of Medical Sciences.*

EDITED BY N. S. DAVIS, M.D., AND F. H. DAVIS, M.D.

No. XI. CHICAGO, JUNE 1, 1874. VOL. XV.

### Original Communications.

### GLEANINGS FROM CAMP AND HOSPITAL.—III.

BY F. K. BAILEY, M.D., KNOXVILLE, TENN.

THERE are thousands living now who will long remember the seven days voyage up the Tennes see river. Leaving Fort Donelson March 4th, two days were consumed going fourteen miles, to a so-called landing, but in reality nothing but a narrow strip of ground between the river and a wide expanse of water, which was almost impassable. Every man on foot was wet to the waist and those on horses were in peril of being thrown off into the mud and mire. Under such circumstances, hundreds of men embarked upon the Belle Memphis, a floating palace in former times, but now anything but desirable. Every spot on the vessel, above and below, from bow to stern, was occupied by either man or beast, with baggage and all the paraphernalia of an army. The weather was cold and damp. Sometimes the mercury went well nigh to 32°, and an opportunity to approach a cheerful fire was seldom enjoyed, except by those who were in command. Day after day passed away and no one can now tell how many were prostrated by a condition of the bowels, ranging from common diarrhœa to a distressing and exhausting inflammation of the mucous lining of the large intestines, known familiarly in the army as flux.*

* NOTE.—Since writing this, I have read a report of Surgeon J. H. Brinton, U. S. V., giving an account of the campaign of the Army of the Tennessee, from February to June, 1862. (Appendix to medical volume, page 24,) in which allusion is made to this bowel affection. He says: "The physical condition of the men about to engage in this severe action was unpromising in the extreme. Many of them had been suffering for weeks, suffering from the diarrhœa peculiar to the

On the 13th, we landed at Savannah, or at least quite a large proportion of the forces, which required over a hundred boats to transport. The first care of the medical officers was to find a house suitable for a regimental hospital. The 20th Illinois selected an abandoned store-house, which was both comfortable and commodious. In a few days not far from ten per cent. of the command were admitted, some of whom were very sick. Every regiment took possession of some building for a hospital, or made use of tents. When the battle of Shiloh occurred, these regimental hospitals were still remaining, and were soon filled to overflowing by wounded men from that memorable field. They were in the care of medical officers who had been left in charge when the army went to the front. It was under those circumstances that I was left behind, instead of being a participant in that terrible conflict which proved so disastrous to our men. My number was increased from about thirty to more than sixty, besides having twelve of Gen. Buell's advance guard, left on Saturday night before the battle.

Before proceeding further, I beg leave to give somewhat in detail a chapter in my own personal history, in connection with our sojourn at Savannah.

As stated in a former number, my health began to suffer during our stay at Fort Donelson. Was able to keep about and attend to the daily round of duties, till the morning of March 30, (Sunday.) About 9 o'clock A. M. a severe chill came on, which compelled me to take the cot. Feeling conscious that it was no ordinary attack, and that a second paroxysm would probably prove fatal, I at once poured out as much quinine as could well be mixed up in a table-spoonful of water, or some other liquid, and swallowed it. There were not less than twenty-five or thirty grains in the dose.

The chill continued until about noon, but no considerable reaction followed. The quinine acted as a powerful sedative, and no pain was experienced.

There was, however, within an hour or two, a sense of extreme fullness in the epigastric and hepatic region. The tension soon became terribly distressing. Before night, I took a few grains of calomel, and followed it with castor-oil and turpentine.

About 9 o'clock, began to feel the effects of the quinine. My cot was placed with its head in the corner of the room. Instead of the usual tinnitus aurium, there was a sensation as if the head would burst, and on closing my eyes, there appeared to be placed perpendicularly in the ceiling, a boiler forty feet long and of a proportionate diameter, filled with men holding their sledges against the heads of rivets, while scores of others upon the outside were hammering with all their might. Those who have visited establishments where steamboat boilers are made, and listened to the deafening sounds of the operation, can imagine what seemed real at this time. This

Tennessee river. This is said to result from the large amount of animal decomposition which takes place on the shoals, a few miles above Pittsburgh Landing. Whether this explanation be correct or not, it is certain that almost every one drinking the waters of the river suffered a profuse diarrhœa, which resisted obstinately the ordinary therapeutic means."

illusion continued till 3 o'clock A. M., before sleep was possible. About midnight the cathartic commenced its operation, and the amount voided was beyond comparison with anything before experienced. Before each evacuation there seemed an antero-posterior slice to be removed from the engaged liver, about an eighth of an inch in thickness. Most of the night passed in this process, which can be described in no better way than above. If I remember rightly, there were no less than twelve or fifteen of those lamina displaced before the mass was removed. When morning came, there was relief from distress, but prostration is a feeble term to employ to express the real condition. There was an intermission of the cranial demonstrations, and no fever came on in the afternoon. At 9 P. M. returned the beating, and the same illusory visions of the previous night. From 3 to 6 there was some sleep, but another fearfully distressed day was passed, followed by a third night of undiminished horror. Sometime on Sunday a medical officer called to ask if Dr. Francis Weaver, of the 45th Illinois infantry, could be brought to my room from his very indifferent quarters in another place. He was told to bring him in by all means, if his condition could thereby be improved. He, too, had been attacked with some grave affection and lay in a mere shed. But he did not come. The doleful funeral dirge, which was played by his escort to the grave, on Wednesday morning, explained his non-appearance. That music was no more pleasing than the hammering of those fancied machinists. During this eventful week, until Friday, my patients up stairs were receiving no attention, except one visit made by my friend, Dr. Harris, of the 53d Illinois. I found on attempting to arise from the cot that there was no power in the spinal muscles. It was impossible to assume the erect posture without assistance. With the aid of a man on each side to lift my weight, I was enabled to go up and attend to the sick. Days and nights passed wearily away till Sunday morning, at an early hour, when we heard a roar of artillery, soon followed by musketry, which ushered in the famous battle of Shiloh. During the night previous Gen. Buell's forces were passing along in front of my room, on their way to the contest; twelve of his advance guard had been tumbled in upon the floor of a room adjoining my own. I was barely able to look at each one without even knowing their condition. Here were more than thirty men in all, with no one able to prescribe for them.

Before Monday night, however, our already crowded rooms were reinforced by an addition of twenty or thirty more, from the battle-field.

By this time I was able, by the help of faithful and willing soldiers, to dress the wounds and prescribe for the sick. For a whole week or more it was necessary to be helped to my bed after sitting down by the bed-side. A strong will, with a tolerably good constitution, enabled me to surmount all the fearful and well-nigh fatal conditions above described. An appetite, such as had not obtained for weeks, soon appeared, and the timely arrival of a fleet of sanitary steamers brought all that heart and stomach could desire. Kind and sympathizing friends also came to cheer the sick and wounded, and to convey such as could be moved, to the well-appointed hospitals in the North.

The number of men who were at Savannah within a week after the battle, has been estimated as high as 2,800. Every available room was filled, besides great numbers were placed in tents.

For two weeks or more I did not go outside of my own division. On looking over the cases received from Buell's command, I found one young man that was laboring under tetanus. Three days before coming in, the great toe of one foot had been crushed by a wheel passing over it.. His comrades reported him sick with mumps at first, but the stiffened jaws were closed by that fearful malady which art seldom relieves. He died on Wednesday, the 9th. His name, as given at the time, was T. H. Parkinson, Co. "F," 19th Ohio. He was not over twenty years old, and a finely formed man. The toe had not been dressed, that we could ascertain, for some days, and the discharges were feted when I first saw the case.

Allusion has been made to the prompt and efficient aid afforded by the arrival of sanitary supplies after the battles of Fort Donelson and Shiloh. Without that source of relief, no tongue can tell what amount of suffering would have been endured. For a soldier in the field, who is well and fit for his duty, the army ration will suffice. Let him become sick or wounded, and more is required, and the kind hearts of those we had left behind us contributed their full share in the cause by following close upon our march with those appliances known as Sanitary Supplies.

And not only were incredible quantities of everything of this character provided, but self-sacrificing men and women left their homes and distributed these articles among the suffering.

On Sunday or Monday, Capt. Cleghorn, of Co. "B," 20th Illinois, was brought in and laid on a cot near my own. A bullet had struck the right arm, anteriorly and near the middle; passing upwards, it encountered the humerus, and caused a comminuted fracture. No place of exit could be found, and the missile was supposed to lie upon the inside a little below the axilla. I removed the dressing which had been applied upon the field, replacing them with others. Two or three surgeons who examined the limb advised amputation, but my idea was that of conservatism. In this opinion the wounded man fully concurred. In a few days he was conveyed to a boat and carried to Cincinnati. On the way, I was subsequently informed, a friend was obliged to stand over him in a threatening attitude to keep him from the knife. He ultimately recovered so as to be able to enter the regular army, where he was, at my latest advices. Capt. North, of Co. "E," 20th Illinois, came in also, with a severe wound upon the right side of the neck. It was painful, and its most interesting feature consisted in the fact that the flying missile avoided the blood-vessels. Lieut. Col. Richards, also, was disabled by a bullet, which struck the buckle of his sword-belt, causing a tumefaction upon the left side of the abdomen, near the crest of the ilium. The shock was severe, and there was apprehension for a time that suppuration would take place. A private had a fore-arm shattered, a spicula of bone wounding one of the arteries. Secondary hemorrhage occurred within a week, and, after trying the persulphate of iron to no effect, the vessel was tied. This was the only operation which was required in No. 4.

